# Elafibranor: a breakthrough therapy revolutionizing primary biliary cholangitis (PBC) treatment

**DOI:** 10.1097/MS9.0000000000002794

**Published:** 2025-01-31

**Authors:** Muhammad H. Gul, Aiman Waheed, Abdul B. Wardak, Yash Shah, Hafsa A. Azam Raja, Badr Ilmaguook, Helai Hussaini, Fatima Zafar

**Affiliations:** a Department of Internal Medicine, Hayatabad Medical Complex, Peshawar, Pakistan; b Department of Internal Medicine,Rawalpindi Medical College, Rawalpindi, Pakistan; c Department of General Surgery,Razia Bahlol Hospital, Kabul, Afghanistan; d Department of Internal Medicine, Trinity Health Oakland, Pontiac, Michigan, USA; e Department of Internal Medicine,Rawalpindi Medical University, Rawalpindi, Pakistan; f Department of Internal Medicine, Wood Hull Medical and Mental Center, Brooklyn, New York, USA; g Department of Internal Medicine, Anaheim Regional Medical Center California, Anaheim, California, USA

The chronic autoimmune a liver condition known as primary biliary cholangitis (PBC) is characterized by the selective killing of tiny intrahepatic biliary epithelial cells. This condition can eventually result in biliary cirrhosis, liver transplantation, or even death. Given that PBC is caused by an organized immune response against the immunodominant mitochondrial autoantigen, the E2 component of the pyruvate dehydrogenase complex (PDC-E2), and is distinguished by the existence of autoreactive CD4 and CD8 T cells and disease-specific antimitochondrial autoantibodies (AMAs), it is regarded as the quintessential autoimmune disease. The pathophysiology of PBC is still mostly unknown, despite a number of significant observations that have significantly improved our understanding of the condition. Despite this, there is strong evidence that the susceptibility of each unique host is determined by a combination of genetic and environmental factors. Environmental factors have been extensively linked over the past few decades to the breakdown of immune tolerance in PBC as a result of epidemiological and genetic research, including genome-wide association studies (GWAS), which have identified important environmental factors and cellular pathways involved in the pathophysiology of the disease[1].

The importance of genetic risk in development of disease is highlighted by several lines of evidence that demonstrate high concordance of disease in monozygotic twins, an increased prevalence in first through fifth degree relatives (FDRs) of affected probands, a sibling relative risk of 10.5, and a high prevalence of AMAs in FDRs compared to population controls. While genetics play an important role, it is likely that allelic variants are not deterministic, but modulate important biologic processes that lead to disease. While significant advances have been made in our understanding of the genetic architecture of PBC, the functional consequences of identified variants and their relevance to important biologic pathways remains undefined[2].

The two most prevalent symptoms among PBC patients are fatigue and pruritus, both of which can considerably reduce quality of life. Excruciating scratching might result in bleeding or excoriations in pruritic patients. In up to 50% of cases, melanin deposition causes skin hyperpigmentation. The condition’s long-term effects include hyperlipidemia, vitamin deficiencies, osteopenia, and osteoporosis. Spider nevi, palmar erythema, ascites, splenomegaly, and muscle atrophy are all classic signs of cirrhosis and portal hypertension. There may also be an increased risk of hepatocellular cancer. It is consequently necessary to have radiographic HCC surveillance during the cirrhotic stage of the disease. Up to 55% of patients with PBC have an additional continuing autoimmune condition, such as autoimmune Hashimoto’s thyroiditis, Sjogren’s syndrome, or Raynaud’s disease. Women are more likely than men to develop PBC[3]. PBC patients usually have a chronic cholestatic biochemical profile, with elevated alkaline phosphatase (ALP) levels to two times or more the upper limit of normal and elevated γ-glutamyl transferase levels to five times the upper limit of normal. These elevated levels are usually maintained for six months or longer. In the early stages of the disease, total bilirubin levels are often normal; however, if they are abnormal, the condition may be worsening. Serum aminotransferases levels may marginally increase. AMA, a highly disease-specific antibody that develops in around 95% of PBC patients, are the serological characteristic of the condition^[4,5]^.

The first-line treatment for PBC is ursodeoxycholic acid (UDCA), as studies have demonstrated a correlation between UDCA-induced reductions in ALP levels and a postponement of liver-related consequences and mortality. But between 15 and 40 percent of individuals have insufficient response to UDCA, or less commonly, they are intolerant[6]. The positive effects of UDCA are mediated by many pathways: (a) choleretic and anti-cholestatic effects, resulting from intracellular molecular signaling pathways that stimulate cellular secretions by promoting vesicular exocytosis and trans-membrane carriers’ insertion; (b) cytoprotection against cytokine-induced injury and the toxic effects of BAs, through stabilization of cell membranes, enhancement of defenses against oxidative stress, and inhibition of apoptosis; additionally, UDCA contributes to the biliary bicarbonate (HCO_3_^−^) umbrella, enhancing biliary HCO_3_^−^ secretion against the acidification of cholangiocytes and hepatocytes due to acid BAs, and up-regulates liver glutathione synthesis; UDCA has several effects, including: (c) immunomodulation and anti-inflammatory effects; by inhibiting prostaglandin E2 (PGE2), it blocks the progression of autoimmune liver injury; additionally, it strongly decreases the hepatocellular expression of MHC class I and the biliary expression of MHC class II, interfering with the autoimmune basic mechanisms; (d) decreases blood levels of eosinophils and suppresses the immune response against pathogen-associate molecular pattern (PAMPs) like lipopolysaccharide’s lipid A[7].

Patients with PBC have recently had elafibranor assessed by FDA. Elafibranor and its active metabolite GFT1007 are PPAR alpha, gamma, and delta agonists that bind to peroxisome proliferator-activated receptors (PPAR). Although the exact method of action of elafibranor is yet unknown, it is believed to suppress the synthesis of bile acids via activating PPAR-alpha and PPAR-delta. The PPAR-delta signaling pathway is responsible for the fibroblast growth factor 21 (FGF21)-dependent downregulation of CYP7A1, the critical enzyme for the generation of bile acids from cholesterol[8]. Moreover, elafibranor decreased the mean levels of ALP in PBC patients. Elafibranor is a dual agonist of the PPAR α and δ, both of which play essential roles in regulating lipid metabolism, glucose homeostasis, and inflammatory pathways. Activation of PPARα stimulates fatty acid oxidation, leading to a reduction in triglyceride levels and an increase in high-density lipoprotein (HDL) cholesterol, improving lipid profiles. Meanwhile, PPARδ activation enhances insulin sensitivity and exerts anti-inflammatory effects by reducing pro-inflammatory cytokine expression. This dual modulation makes Elafibranor particularly effective in treating conditions such as nonalcoholic steatohepatitis (NASH) and PBC, where disrupted metabolic processes and chronic inflammation are central to disease progression.

Elafibranor’s ability to target multiple metabolic and inflammatory pathways simultaneously offers a broader therapeutic approach compared to traditional treatments that often focus on singular disease mechanisms. Its favorable safety profile, coupled with its ability to reduce disease markers like ALP and inflammation-related proteins, positions it as a promising option for managing complex metabolic and liver disorders. As stated by Kowdley *et al*, in an international, phase 3, double-blind, placebo-controlled study, patients with PBC who had not responded well to UDCA or had unacceptable side effects were randomized (2:1) to receive a placebo or 80 mg of elafibranor once daily. The primary result was a biochemical response, which was defined as normal total bilirubin levels and an ALP level at week 52 that was less than 1.67 times the upper limit of the normal range and had dropped by at least 15% from baseline. Secondary end outcomes included normalization of ALP levels at week 52 and a change in pruritus severity from baseline to week 52 and week 24, as determined by the Worst Itch Numeric Rating Scale (WI-NRS; scores range from 0 [no itch] to 10 [worst itch possible]). A total of 161 patients were randomized. A biochemical response (the major end point) was recorded in 51% of the patients (55 of 108) who got elafibranor and in 4% (2 of 53) who received placebo, with a difference of 47 percentage points (95% confidence interval [CI], 32–57; *P* < 0.001). At week 52, 15% of the patients in the elafibranor group and none of the patients in the placebo group had normalized ALP levels (difference, 15 percentage points; 95% confidence interval, 6–23; *P* = 0.002). Among patients with moderate-to-severe pruritus (44 in the elafibranor group and 22 in the placebo group), the least-squares mean change from baseline to week 52 on the WI-NRS did not differ significantly between the groups (−1.93 vs. −1.15, difference, −0.78; 95% CI, −1.99 to 0.42; *P* = 0.20). Abdominal discomfort, diarrhea, nausea, and vomiting were the most common adverse events reported with elafibranor compared to placebo[9]. The summary of clinical trial is given in Table 1.

**Table 1:** Efficacy and safety of elafibranor in primary biliary cholangitis

Outcome measure	Elafibranor group	Placebo group	Difference (95% CI)
Biochemical response (week 52)	51%	4%	47% (32–57)
Alkaline phosphatase normalization (week 52)	15%	0%	15% (6–23)
Pruritus intensity change (WI-NRS)	No significant difference	−1.93 (Elafibranor) vs. −1.15 (Placebo)	−0.78 (−1.99 to 0.42)

Biochemical response: Defined as an alkaline phosphatase level < 1.67 times the upper limit of the normal range, with a reduction of ≥15% from baseline, and normal total bilirubin levels.

Alkaline phosphatase normalization: Percentage of patients with normalized alkaline phosphatase levels.

Pruritus intensity change: Measured using the Worst Itch Numeric Rating Scale (WI-NRS).

In agreement with Caiou *et al*, this 12-week, double-blind phase II trial included 45 persons with PBC who had an incomplete response to UDCA (ALP levels > −1.67-fold the upper limit of normal). Patients were randomly randomized to receive elafibranor 80 mg, elafibranor 120 mg, or a placebo. The primary outcome was the relative change in ALP during 12 weeks (NCT03124108). At 12 weeks, elafibranor 80 mg lowered ALP by −48.3 ± 14.8% (*P* < 0.001), whereas placebo increased it by +3.2 ± 14.8%. The composite endpoint of ALP < −1.67-fold the ULN, decrease of ALP > 15%, and total bilirubin below the ULN was achieved in 67% of patients in the elafibranor 80 mg group and 79% in the elafibranor 120 mg group, compared to 6.7% in the placebo group. The gamma-glutamyltransferase levels fell by 37.0 ± 25.5% in the elafibranor 80 mg group (*P* < 0.001) and 40.0 ± 24.1% in the elafibranor 120 mg group (*P* < 0.01), whereas the placebo group showed no change (+0.2 ± 26.0%). Elafibranor also lowered the levels of illness indicators such as IgM, 5ʹ-nucleotidase, and high-sensitivity C-reactive protein. Elafibranor did not induce or exacerbate pruritus, and by the time the treatment was over, those who experienced pruritus at the beginning reported having less of it. Every probable non-serious side effect associated with the medication was mild to moderate, with the most common being digestive disorders such as nausea and diarrhea. Importantly, no significant increases in adverse effects were reported between the treated group and the placebo group. In terms of safety, the trial reported that Elafibranor (GFT505) was well tolerated. Liver function tests showed no signs of hepatotoxicity, a crucial aspect when considering the safety of PPAR agonists. Overall, Elafibranor demonstrated a favorable safety profile with no serious adverse events related to the treatment, supporting its further clinical development. Elafibranor significantly reduced levels of ALP, composite endpoints of bilirubin and ALP, as well as other markers of disease activity in patients with PBC who did not respond fully to UDCA. It was also generally safe and well tolerated in this randomized phase II trial.[10]. A network meta-analysis conducted by Lin W et al was applicable to a total of 23 papers. The following combinations of UDCA and ALP improved ALP levels more than UDCA monotherapy: bezafibrate (MD 104.49, 95% CI 60.41, 161.92), fenofibrate (MD 87.81, 95% CI 52.34, 129.79), OCA (MD 65.21, 95% CI 8.99, 121.80), seladelpar (MD 117.39, 95% CI 19.97, 213.95), elafibranor (MD 140.73, 95% CI 74.34, 209.98), and saroglitazar (MD 132.09, 95% CI 13.99, 247.04) combined with UDCA. The combination of elafibranor and UDCA was most likely (32%) to be the best possible medication combination[11].

With its promising efficacy and safety profile, elafibranor marks a major breakthrough in the treatment of PBC. Patients who are not responding to conventional medicines now have a new therapeutic option thanks to its distinct method of action. Elafibranor has the potential to transform PBC management as long as continuous research supports its advantages.

## Data Availability

Not applicable.
